# GABA_B_ Receptor-Mediated Impairment of Intermediate Progenitor Maturation During Postnatal Hippocampal Neurogenesis of Newborn Rats

**DOI:** 10.3389/fncel.2021.651072

**Published:** 2021-08-06

**Authors:** Charlotte Gustorff, Till Scheuer, Thomas Schmitz, Christoph Bührer, Stefanie Endesfelder

**Affiliations:** Department of Neonatology, Charité—Universitätsmedizin Berlin, Berlin, Germany

**Keywords:** GABA, postnatal neurogenesis, brain development, hippocampus, rat—brain

## Abstract

The neurotransmitter GABA and its receptors assume essential functions during fetal and postnatal brain development. The last trimester of a human pregnancy and early postnatal life involves a vulnerable period of brain development. In the second half of gestation, there is a developmental shift from depolarizing to hyperpolarizing in the GABAergic system, which might be disturbed by preterm birth. Alterations of the postnatal GABA shift are associated with several neurodevelopmental disorders. In this *in vivo* study, we investigated neurogenesis in the dentate gyrus (DG) in response to daily administration of pharmacological GABA_A_ (DMCM) and GABA_B_ (CGP 35348) receptor inhibitors to newborn rats. Six-day-old Wistar rats (P6) were daily injected (i.p.) to postnatal day 11 (P11) with DMCM, CGP 35348, or vehicle to determine the effects of both antagonists on postnatal neurogenesis. Due to GABA_B_ receptor blockade by CGP 35348, immunohistochemistry revealed a decrease in the number of NeuroD1 positive intermediate progenitor cells and a reduction of proliferative Nestin-positive neuronal stem cells at the DG. The impairment of hippocampal neurogenesis at this stage of differentiation is in line with a significantly decreased RNA expression of the transcription factors *Pax6*, *Ascl1*, and *NeuroD1*. Interestingly, the number of NeuN-positive postmitotic neurons was not affected by GABA_B_ receptor blockade, although strictly associated transcription factors for postmitotic neurons, *Tbr1*, *Prox1*, and *NeuroD2*, displayed reduced expression levels, suggesting impairment by GABA_B_ receptor antagonization at this stage of neurogenesis. Antagonization of GABA_B_ receptors decreased the expression of neurotrophins* (BDNF*, *NT-3*, and *NGF)*. In contrast to the GABA_B_ receptor blockade, the GABA_A_ receptor antagonization revealed no significant changes in cell counts, but an increased transcriptional expression of *Tbr1* and *Tbr2*. We conclude that GABAergic signaling *via* the metabotropic GABA_B_ receptor is crucial for hippocampal neurogenesis at the time of rapid brain growth and of the postnatal GABA shift. Differentiation and proliferation of intermediate progenitor cells are dependent on GABA. These insights become more pertinent in preterm infants whose developing brains are prematurely exposed to spostnatal stress and predisposed to poor neurodevelopmental disorders, possibly as sequelae of early disruption in GABAergic signaling.

## Introduction

Very preterm-born children may suffer from significant deficits in executive function, processing speed, and intelligence (Brydges et al., 2018). The risk for neurodevelopmental disorders such as attention-deficit/hyperactivity disorder (ADHD), autism spectrum disorder (ASD), and anxiety are increased two to four times in preterm children, as compared to term-born controls (Rogers et al., 2018). Altered GABAergic signaling has been implicated in the pathogenesis of ASD (Gaetz et al., 2014; Tanifuji et al., 2017), ADHD (Naaijen et al., 2017), as well as anxiety (Nuss, 2015). Pharmacological antagonization and agonization of γ-aminobutyric acid (GABA) receptors in the neonatal animal model of the mouse during postnatal brain development corroborate an important role of GABA and their receptors in programming neurobehavioral phenotypes in adulthood (Salari and Amani, 2017).

While human brain growth reaches its highest velocity at birth (Watson et al., 2006), the so-called “brain growth spurts” in rodents peak at the seventh postnatal day (Semple et al., 2013). Postnatal rat pups may therefore serve as a model for the human stage of brain development, corresponding to the last trimester of pregnancy. Both in humans and rodents, the hippocampus undergoes developmental changes close to birth (Semple et al., 2013). During the formation of the dentate gyrus (DG) neural progenitor cells (NPC) are generated and form a proliferative zone that remains active during postnatal stages, becoming the site of adult hippocampal neurogenesis called the subgranular zone (SGZ; Paridaen and Huttner, 2014). Supplemented new neurons form highly complex neural circuits, supporting a role for hippocampal neurogenesis in memory, learning, and behavior (Deng et al., 2010; Anacker and Hen, 2017).

The last trimester of a human pregnancy and early postnatal life involves a period of brain development with neuronal organization and maturation, such as neurogenesis, migration, dendritogenesis, synaptogenesis, and plasticity of developing neurons. These processes are regulated by neurotransmitters such as GABA and glutamate, which remain at risk of disruption after preterm birth (Malik et al., 2013). In humans, the phase of rapid brain growth starts at gestational week 28, and peaks at the time of birth. In the rat model, this phase occurs from postnatal day (P)4 to P11 and peaks at P7 (Dobbing and Sands, 1979; Semple et al., 2013) which makes early postnatal pups useful as model organisms in studies of human neuronal development, corresponding roughly to the last trimester of pregnancy. These neurodevelopmental processes are highly vulnerable and clinically relevant, as they can be affected by oxidative stress (hyperoxia, hypoxia) or various necessary medical interventions (Malik et al., 2013; Steinhorn et al., 2015; Duerden et al., 2016; Isokawa, 2016). In addition to neurotransmission and developmentally mediated excitatory-inhibitory transition of GABA action during the perinatal period (Ben-Ari, 2018), GABA and its receptors assume multiple essential functions during fetal and postnatal brain development (Cellot and Cherubini, 2013; Wu and Sun, 2015; Tang et al., 2021). In the rat, the main generation of hippocampal granular cells starts around birth and peaks during the first postnatal week (Altman and Bayer, 1990). In line with this, GABAergic transmission changes from excitatory to inhibitory also during the end of the first postnatal week (Rivera et al., 1999; Khirug et al., 2005). Neurobehavioral disorders, including autism, are more common in survivors of preterm birth and have been associated with decreased GABA concentrations, underscoring the importance of* in vivo* examination of GABA changes during early postnatal life in preterm infants (Ream and Lehwald, 2018; Basu et al., 2021). Peerboom and Wierenga (2021) postulated that the postnatal shift from depolarizing to hyperpolarizing GABA is a pivotal event in brain development and its timing affects brain function throughout life. Altered timing of the postnatal GABA shift is associated with several neurodevelopmental disorders (Schulte et al., 2018). In addition, preterm birth itself, as well as pharmacologic drugs used in the preterm infant, influence GABA receptor associated pathways (Shaw et al., 2015; Steinhorn et al., 2015). Extremely and very preterm infants showed reduced GABA concentrations in the brain measured by magnetic resonance imaging (Kwon et al., 2014; Basu et al., 2020). The developmental timeline of the GABAergic system becomes more relevant in preterm infants whose developing brains are prematurely exposed to extra uterine stress, and predisposed to neurological disorders, perhaps in part as sequela of early derangement in GABAergic systems.

Despite the postnatal developmental differences in GABAergic signaling, there are many similarities in the generalized course of neural maturation in early development and adulthood (Song et al., 2012). A complex interaction of the intrinsic programs of neuronal stem cells (NSCs) and their progressively produced progenitors (NPC) regulates neurogenesis, which is orchestrated by intrinsic pathways and extracellular signaling molecules (Faigle and Song, 2013; Bjornsson et al., 2015). As shown schematically in Figure 5, hippocampal neurogenesis originates from NPC and leads to granule cell neurons, which go through different stages leads to granule cell neurons after progressing through the stages of NSC/type-1 cells, NPC/type-2a cells, neuroblast/type-2b cells, immature-mitotic neuron/type-3 cells as well as postmitotic-immature and mature granular neurons (Kempermann et al., 2004). NSCs are the shared NPC of both neurons and astrocytes and therefore express the astrocytic markers glial fibrillary acidic protein (GFAP) and Scl1a3 as well as the neuronal marker nestin (DeCarolis et al., 2013; Berg et al., 2018; Vieira et al., 2018). NSCs express Sox2 to maintain their multipotency and proliferation capacity (Mercurio et al., 2019). Sox2 represses the expression of NeuroD1 and therefore prevents the cells’ progression in neurogenesis, preserving their self-renewal capacity (Kuwabara et al., 2009). A key regulator of the mainstay of NSCs after their transition to asymmetric neurogenic division is the Notch target gene Hes5 (Lugert et al., 2010). By directly changing the expression of genes associated with self-renewal and differentiation [e.g., Sox2 (Wen et al., 2008), Ngn2, and NeuroD1 (Scardigli et al., 2003; Shimojo et al., 2008)], Pax6 is essential to regulating the NPCs’ proliferation (Maekawa et al., 2005). NSCs give rise to intermediate progenitor cells, which express as a specific marker Tbr2. Tbr2 labels type-2 cells, while Tbr1 is expressed by immature granule neurons (Englund et al., 2005; Hodge et al., 2008; Nicola et al., 2015). Additionally, type-2a cells express the proneural markers Ascl1 (also known as Mash1) and Ngn2 (Amador-Arjona et al., 2015; Pérez-Domínguez et al., 2018). At the late stage of typ-2a cells, Ngn2 is downregulated, whilst Tbr2 expression in typ-2b cells persists (Roybon et al., 2009). Type-2b cells start to express NeuroD1, a crucial transcription factor for neurogenesis during hippocampal development that marks the transition from amplifying progenitor to neuroblast (Kuwabara et al., 2009). NeuroD1 is necessary for further survival and maturation of neurons (Pérez-Domínguez et al., 2018). Type-3 cells are less proliferative and may migrate and exit the cell cycle before full maturation into granule neurons (Nicola et al., 2015). NeuroD2 starts to be expressed just after NeuroD1 and continues to be highly expressed in postmitotic mature neurons (Roybon et al., 2009). Postmitotic neurons following further differentiation express as specific markers NeuN, Tbr1 (Englund et al., 2005) and NeuroD2, of which the latter is necessary for cell cycle regulation and survival of neurons (Olson et al., 2001; Wilke et al., 2012). Granule cell maturation necessarily depends on Prox1 expression, which starts with type-2b cells and is maintained further in differentiation (Lavado et al., 2010). Different types of signals, including glutamatergic and GABAergic signals from local neural networks, mediate these complex neurogenic processes.

**Figure 1:**
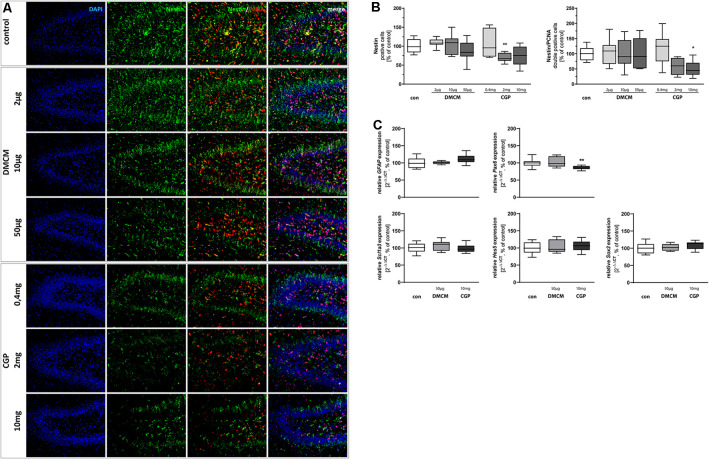
Representative hippocampal paraffin sections **(A)** of control animals, DMCM hydrochloride (DMCM) in doses of either 2 μg/kg, 10 μg/kg or 50 μg/kg, and CGP 35348 in doses of either 0.4 mg/kg, 2 mg/kg or 10 mg/kg treated rat pups at P11 co-labeled with DAPI, Nestin, and PCNA. Application of GABA_B_ receptor antagonist CGP 2 mg/kg decreased Nestin positive progenitor cells in the dentate gyrus (DG). Application of CGP 10 mg/kg decreased the number of proliferating Nestin/PCNA double positive cells. Quantification of **(B)** Nestin and Nestin/PCNA double positive cells in sum of the DG in comparison to control group (100% white bars). Data are expressed relative to the control group as mean ± SEM of *n* = 10 each group. The 100% values are for Nestin+ 83.5 cell counts and for Nestin+PCNA+ 13.3 cell counts. *p < 0.05 and **p < 0.01 vs. control (Brown-Forsythe test for Nestin+, Kruskal–Wallis test for Nestin+PCNA+). Expressions of **(C)** *glial fibrillary acidic protein (GFAP)*, *Scl1a3*, *Hes5*, and *Sox2* are not affected by the application of DMCM or CGP. *Pax6* expression is diminished in CGP treated animals. The relative mRNA expressions of markers were measured by quantitative real-time PCR in rat brain homogenates with DMCM 50 μg/kg (gray bars) or CGP 10 mg/kg (black bars) application relative to control (white bars). Bars represent the relative mRNA quantification based on internal standard *HPRT*. Data shown as mean ± SEM, *n* = 9–10. **p < 0.01 vs. control (Brown-Forsythe test for *GFAP*, one-way analysis of variance (ANOVA) for *Scl1a3*, *Hes5*, *Sox2*, and *Pax6*).

**Figure 2:**
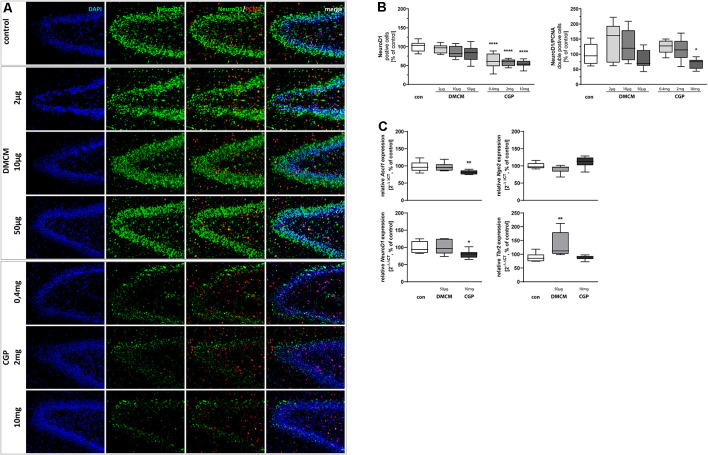
Representative hippocampal paraffin sections **(A)** of control animals, DMCM hydrochloride (DMCM) in doses of either 2 μg/kg, 10 μg/kg or 50 μg/kg, and CGP 35348 in doses of either 0.4 mg/kg, 2 mg/kg or 10 mg/kg treated rat pups at P11 co-labeled with DAPI, NeuroD1, and PCNA. Application of GABA_B_ receptor antagonist CGP decreased NeuroD1 positive progenitor cells in the DG and NeuroD1/PCNA double positive cells in the group with the highest dose of 10 mg/kg. Quantification of **(B)** NeuroD1 and NeuroD1/PCNA double positive cells in sum of the DG in comparison to control group (100% white bars). Data are expressed relative to the control group as mean ± SEM of *n* = 10 each group. The 100% values are for NeuroD1+ 308.9 cell counts and for NeuroD1+PCNA+ 7.7 cell counts. *p < 0.05 and ****p < 0.0001 vs. control (one-way ANOVA for NeuroD1+, Brown-Forsythe test for NeuroD1+PCNA+). Expressions of **(C)** *Ascl1* and *NeuroD1* are reduced in CGP treated animals and expression of *Tbr2* is increased in DMCM treated animals. *Ngn2* does not get affected by GABA receptor antagonists. The relative mRNA expressions of markers were measured by quantitative real-time PCR in rat brain homogenates with DMCM 50 μg/kg (gray bars) or CGP 10 mg/kg (black bars) application relative to control (white bars). Bars represent the relative mRNA quantification based on internal standard *HPRT*. Data shown as mean ± SEM, *n* = 9–10. *p < 0.05 and **p < 0.01 vs. control (one-way ANOVA for *Ascl1* and *Tbr2*, Kruskal–Wallis test for *NeuroD1*, Brown-Forsythe test for *Ngn2*).

**Figure 3:**
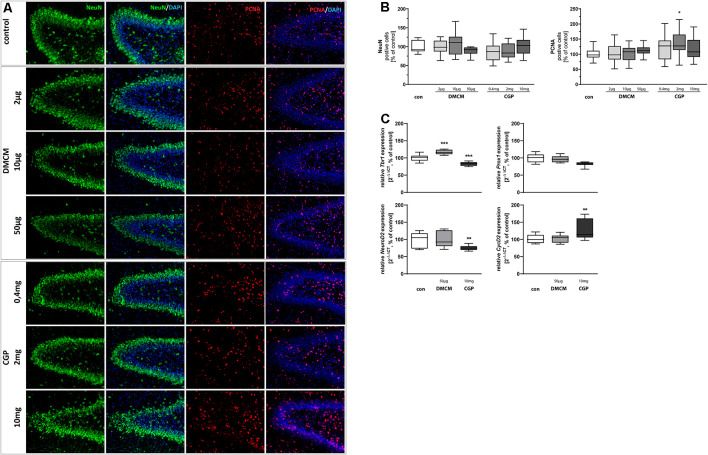
Representative hippocampal paraffin sections **(A)** of control animals, DMCM hydrochloride (DMCM) in doses of either 2 μg/kg, 10 μg/kg or 50 μg/kg, and CGP 35348 in doses of either 0.4 mg/kg, 2 mg/kg or 10 mg/kg treated rat pups at P11 co-labeled with DAPI, NeuN, and PCNA. Application of GABA receptor antagonists did not affect cell counts for postmitotic NeuN+ neurons at the DG. Application of CGP 2 mg/kg led to an increased number of proliferating PCNA+ cells. Quantification of **(B)** NeuN and PCNA positive cells in sum of the DG in comparison to control group (100% white bars). Data are expressed relative to the control group as mean ± SEM of *n* = 10 each group. The 100% values are for NeuN+ 143.0 cell counts and for PCNA+ 81.8 cell counts. *p < 0.05 vs. control (Brown-Forsythe test). Expressions of **(C)** *Tbr1* and *NeuroD2* are reduced and expression of *CycD2* is increased in CGP treated animals. Expression of *Tbr1* is increased in DMCM treated animals. GABA receptor antagonists do not affect the expression of *Prox1*. The relative mRNA expressions of markers were measured by quantitative real-time PCR in rat brain homogenates with DMCM 50 μg/kg (gray bars) or CGP 10 mg/kg (black bars) application relative to control (white bars). Bars represent the relative mRNA quantification based on internal standard *HPRT*. Data shown as mean ± SEM, *n* = 9–10. **p < 0.01 and ***p < 0.001 vs. control (Brown-Forsythe test for *CycD2* and *NeuroD2*, one-way ANOVA for *Tbr1*).

**Figure 4:**
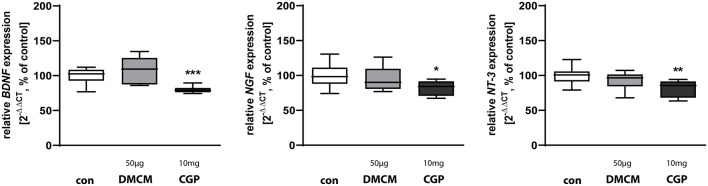
Expression of neurotrophins *BDNF*, *NGF*, and *NT-3* is reduced in CGP treated animals. The relative mRNA expression of markers was measured by quantitative real-time PCR in rat brain homogenates with DMCM 50 μg/kg (gray bars) or CGP 10 mg/kg (black bars) application relative to control (white bars). Bars represent the relative mRNA quantification based on internal standard *HPRT*. Data shown as mean ± SEM, *n* = 9–10. *p < 0.05, **p < 0.01, and ***p < 0.001 vs. control (Brown-Forsythe test for *BDNF*, one-way ANOVA for *NGF* and *NT-3*).

**Figure 5:**
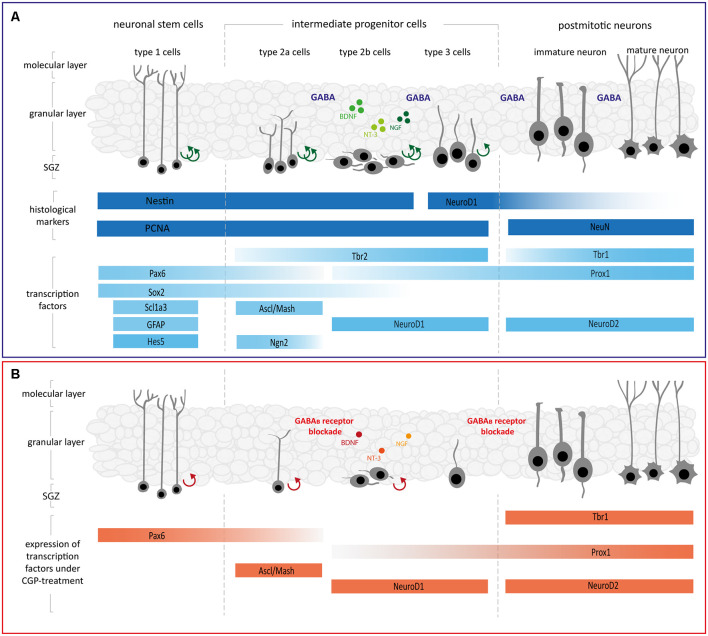
**(A)** Schematic diagram illustrating the different phases of neurogenesis in the DG. Asymmetrically dividing neuronal stem cells situated at the subgranular zone (SGZ) of the DG give rise to intermediate progenitor cells type-2a, type-2b, and type-3, which are highly proliferative, and their progeny determined for neuronal fate. Following further differentiation, intermediate progenitors extend processes, mature, exit the cell cycle and finally differentiate into mature neurons. Most of the regulators play an important role in self-renewal, proliferation, and fate specification during neurogenesis (see main text for details, modeled after Roybon et al., 2009; Lugert et al., 2010; Berg et al., 2018; Vieira et al., 2018; Hevner, 2019). **(B)** Schematic diagram depicting how the GABA_B_ receptor blockade changes single steps of hippocampal neurogenesis. GABA_B_ receptor blockade with CGP led to reductions in proliferating Nestin+ as well as NeuroD1+ cells, furthermore, expression of *Pax6, Ascl1, NeuroD1, Tbr1, Prox1*, and *NeuroD2* was decreased (see main text for details, modeled after Roybon et al., 2009; Lugert et al., 2010; Berg et al., 2018; Vieira et al., 2018; Hevner, 2019). Abbreviations: Ascl1, achaete-scute family bHLH transcription factor 1; BDNF, brain-derived neurotrophic factor; CycD2, cyclin D2; GABA, γ-aminobutyric acid; GFAP, glial fibrillary acidic protein; Hes5, hairy-enhancer-of-split 5; NeuroD1/2, neurogenic differentiation 1/ 2; Ngn2, neurogenin 2; NGF, nerve growth factor; NT-3, neurotrophin 3; Pax6, paired box 6; Prox1, prospero homeobox 1; Sox2, SRY-box transcription factor 2; Scl1a3, solute carrier family 1 member 3; SGZ, subgranule zone; Tbr1/2, T-box brain transcription factor 1/2.

The neurotransmitter GABA, in tandem with the neurotransmitter glutamate and its receptors, is essential for balancing excitation and inhibition and subject to various changes during pre- and postnatal development (Takesian and Hensch, 2013). The ionotropic GABA_A_ receptor and the metabotropic GABA_B_ receptor are two main subtypes of GABA receptors. Berg et al. (2013) postulated that in addition to the progression of neural stem cells into functionally integrated mature neurons, cell cycle regulation and cell differentiation might be part of the functions of neurotransmitters. Various neurotransmitter receptors are expressed on different neuronal cell types (Pocock and Kettenmann, 2007). The functional roles for GABA during adult neurogenesis are already well described in part (Ge et al., 2006; Giachino et al., 2014; Song et al., 2016; Catavero et al., 2018), but it is poorly understood which steps in hippocampal neurogenesis at the time of brain growth spurts are vulnerable to disturbance of GABAergic signaling. Since the referenced evidence suggests that modulation of the GABA receptors during early life induces behavioral abnormalities in later life, we hypothesized that neonatal pharmacological blockade of GABA_A_ and GABA_B_ receptors during excitatory to inhibitory switch of GABA signaling may alter neuronal proliferation, differentiation, and maturation of hippocampal neurons in newborn rat pups.

## Materials and Methods

### Animal Welfare

Time-pregnant Wistar rat dams were obtained from the Department of Experimental Medicine (FEM, Charité—Universitätsmedizin Berlin, Germany). The rat litters were housed with lactating mother under temperature- and humidity-controlled 12 h/12 h light/dark cycle conditions with *ad libitum* access to food and water. All animal experimental procedures were approved by the local animal welfare authorities (LAGeSo, approval number G-0075/18) and followed institutional guidelines as well as ARRIVE guidelines.

### Drug Administration

Rat pups were cross-gender randomly assigned into a control group with 0.9% saline and six verum groups with GABA_A_ receptor antagonist DMCM (4-Ethyl-6,7-dimethoxy-9H-pyrido[3,4-b]indole-3-carboxylic acid methyl ester) hydrochloride administered at three dosages (2 μg/kg, 10 μg/kg, or 50 μg/kg body weight; Tocris, cat. no. 3083, Wiesbaden-Nordenstadt, Germany), and with GABA_B_ receptor antagonist CPG 35348 at 0.4 mg/kg, 2 mg/kg, or 10 mg/kg body weight (Tocris, cat. no. 1245), respectively. Beginning at postnatal day 6 (P6) the rat pups were intraperitoneally injected (i.p.) with saline, DMCM, or CGP 35348 daily on six consecutive days (P6 to P11) with a weight-adapted volume of 0.1 ml per 10 g of body weight. The dosage of the two GABA receptor antagonists was selected to exclude seizures and shakiness. Behavioral seizure responses were monitored for 30 min after drug administration. No pups died or scored for seizure activity. Animals were sacrificed within 12 h following the last injection. For dose-dependent histological analysis of GABA receptor antagonization, each group entailed 10 animals. Gene expression analyses were done in animals receiving the highest concentration of GABA receptor antagonists (DMCM hydrochloride 50 μg/kg and CGP 35348 10 mg/kg body weight (i.p.), respectively) and included nine to 10 animals. The two substances used are hereinafter referred to as DMCM and CGP.

### Tissue Preparation

For histological analysis, as previously described (Endesfelder et al., 2018), at postnatal day 11 (P11) rat pups were transcardially perfused with ice-cold phosphate-buffered saline (PBS, pH 7.4), followed by 4% paraformaldehyde (PFA) in PBS under anesthesia of ketamine (100 mg/kg), xylazine (20 mg/kg), and acepromazine (3 mg/kg). The removed brain tissues were post-fixed in 4% PFA at 4°C for 24 h. Afterward they were transferred to PBS (pH 7.4) and stored at 4°C until paraffin embedding. In preparation for immunohistochemical analyses, the brains were embedded in paraffin. For this purpose, the tissues were first washed under running water for 4 h. This was followed by various dehydrating incubations of the brain tissue with increasing alcohol (ethanol) concentrations (70% for 4 h and 80% overnight at room temperature, 96% and two changes of 100% for each 1 h at 40°C) and chloroform (100% ethanol/chloroform (1:1) and two changes of chloroform for each 1 h at 40°C). Finally, the tissues were immersed in two changes of paraffin (1 h and overnight) at 60°C, cooled, and stored at room temperature.

For gene expression analysis, animals were transcardially perfused with ice-cold PBS (pH 7.4). After decapitation, the olfactory bulb and cerebellum were removed, and brain hemispheres were snap-frozen in liquid nitrogen and stored at −80°C.

### RNA Extraction and Quantitative Real-Time PCR

The gene expression analysis was performed as previously described (Endesfelder et al., 2020). In short, total RNA was isolated from frozen tissue of the whole hemisphere by acidic phenol/chloroform-extraction (peqGOLD RNAPure^TM^; PEQLAB Biotechnologie, cat. no. 30-1010, Erlangen, Germany). 2 μg of DNase-treated RNA was reverse transcribed. In real time the PCR products of the following genes were quantified: achaete-scute family bHLH transcription factor 1 (*Ascl1*), brain-derived neurotrophic factor (*BDNF*), cyclin D2 (*CycD2*), GFAP, hairy-enhancer-of-split 5 (*Hes5*), hypoxanthine-guanine phosphoribosyl-transferase (*HPRT*), neurogenic differentiation 1 (*NeuroD1*), neurogenic differentiation 2 (*NeuroD2*), neurogenin 2 (*Ngn2*), nerve growth factor (*NGF*), neurotrophin 3 (*NT-3*), paired box 6 (*Pax6*), prospero homeobox 1 (*Prox1*), SRY-box transcription factor 2 (*Sox2*), solute carrier family 1 member 3 (*Scl1a3*), T-box brain transcription factor 1 (*Tbr1*), and T-box brain transcription factor 2 (*Tbr2*).

Table 1 shows the sequences of dye-labeled fluorogenic reporter oligonucleotides used for real time amplification. Probes were labeled with the fluorescent reporter 6-carboxy-fluorescein (6-FAM) at the 5′ end and the fluorescent quencher carboxytetramethylrhodamine (TAMRA) at the 3′ end. PCR and detection were performed with qPCR BIO Mix Hi-ROX (NIPPON Genetics Europe, cat. no. PB20.22-51, Düren, Germany) with *HPRT* used as an internal reference. The expression of target genes was analyzed according to the 2^−ΔΔCT^ method (Livak and Schmittgen, 2001) with the StepOnePlus real-time PCR system (Applied Biosystems/Life Technologies, Carlsbad, CA, USA).

**Table 1:** Sequences of oligonucleotides.

	Oligonucleotide sequence 5′-3′	Accession No.
***AIF***		
forward	CACAAAGACACTGCAGTTCAGACA	NM_031356.1
reverse	AGGTCCTGAGCAGAGACATAGAAAG
probe	AGAAGCATCTATTTCCAGCC
***Ascl1 (Mash1)***		
forward	AACTTCAGTGGCTTCGGCTA	NM_022384.1
reverse	GCCCAGGTTAACCAACTTGA
probe	AGCCTTCCACAGCAGCAG
***BDNF***		
forward	TCAGCAGTCAAGTGCCTTTGG	NM_012513.4
reverse	CGCCGAACCCTCATAGACATG	
probe	CCTCCTCTGCTCTTTCTGCTGGAGGAATACAA	
***Casp3***		
forward	ACAGTGGAACTGACGATGATATGG	NM_012922.2
reverse	AATAGTAACCGGGTGCGGTAGA	
probe	ATGCCAGAAGATACCAGTGG	
***CycD2***		
forward	CGTACATGCGCAGGATGGT	NM_199501.1
reverse	AATTCATGGCCAGAGGAAAGAC	
probe	TGGATGCTAGAGGTCTGTGA	
***GFAP***		
forward	TCTGGACCAGCTTACTACCAACAG	NM_017009.2
reverse	TGGTTTCATCTTGGAGCTTCTG	
probe	AGAGGGACAATCTCACACAG	
***Hes5***		
forward	ATGCTCAGTCCCAAGGAGAA	NM_024383.1
reverse	TAGTCCTGGTGCAGGCTCTT	
probe	CCCAACTCCAAACTGGAGAA	
***HPRT***		
forward	GGAAAGAACGTCTTGATTGTTGAA	NM_012583.2
reverse	CCAACACTTCGAGAGGTCCTTTT	
probe	CTTTCCTTGGTCAAGCAGTACAGCCCC	
***NeuroD1***		
forward	TCAGCATCAATGGCAACTTC	NM_019218.2
reverse	AAGATTGATCCGTGGCTTTG	
probe	TTACCATGCACTACCCTGCA	
***NeuroD2***		
forward	TCTGGTGTCCTACGTGCAGA	NM_019326.1
reverse	CCTGCTCCGTGAGGAAGTTA	
probe	TGCCTGCAGCTGAACTCTC	
***Ngn2***		
forward	AGGCTCAAAGCCAACAACC	XM_008775262.2
reverse	GATGTAATTGTGGGCGAAGC	
probe	CTCACGAAGATCGAGACGCT	
***NGF***		
forward	ACCCAAGCTCACCTCAGTGTCT	NM_001277055.1
reverse	GACATTACGCTATGCACCTCAGAGT	
probe	CAATAAAGGCTTTGCCAAGG	
***NT-3***		
forward	AGAACATCACCACGGAGGAAA	NM_031073.3
reverse	GGTCACCCACAGGCTCTCA	
probe	AGAGCATAAGAGTCACCGAG	
***Pax6***		
forward	TCCCTATCAGCAGCAGTTTCAGT	NM_013001.2
reverse	GTCTGTGCGGCCCAACAT	
probe	CTCCTCCTTTACATCGGGTT	
***Prox1***		
forward	TGCCTTTTCCAGGAGCAACTAT	NM_001107201.1
reverse	CCGCTGGCTTGGAAACTG	
probe	ACATGAACAAAAACGGTGGC	
***Sox2***		
forward	ACAGATGCAGCCGATGCA	NM_001109181.1
reverse	GGTGCCCTGCTGCGAGTA	
probe	CAGTACAACTCCATGACCAG	
***Scl1a3 (GLAST)***		
forward	CCCTGCCCATCACTTTCAAG	NM_001289942.1
reverse	GCGGTCCCATCCATGTTAA	
probe	CTGGAAGAAAACAATGGTGTGG	
***Tbr1***		
forward	TCCCAATCACTGGAGGTTTCA	NM_001191070.1
reverse	GGATGCATATAGACCCGGTTTC	
probe	AAATGGGTTCCTTGTGGCAA	
***Tbr2***		
forward	ACGCAGATGATAGTGTTGCAGTCT	XM_006226608.2
reverse	ATTCAAGTCCTCCACACCATCCT	
probe	CACAAATACCAACCTCGACT	

### Immunohistochemistry

Paraffin-embedded coronal sections of the brains were serially cut into 5 μm sections and mounted onto Super Frost Plus-coated slides (Menzel, Braunschweig, Germany). The sections were deparaffinized in Roti-Histol twice for 10 min each. The PFA-fixed tissues were dehydrated through incubation in aqueous solutions of decreasing ethanol concentration. The slices were immersed for 3 min each in ethanol (100%, 100%, 90%, 80%, 70%). To demask intracellular epitopes, sections were fixed in citrate buffer (pH 6.0) in a microwave oven for 10 min at 600 W. The sections then were cooled down at room temperature for 30 min and were washed three times afterward in PBS for NeuN/DAPI- and Nestin/PCNA/DAPI-staining and in Tris-buffered saline (TBS) for NeuroD1/PCNA/DAPI-staining. For NeuN/DAPI- and Nestin/PCNA/DAPI-staining blocking solution [3% bovine serum albumin (BSA), 0.2% TX-100 in PBS] was applicated to each section for 60 min. For NeuroD1/PCNA/DAPI staining, blocking solution (5%BSA, 0.5% TX-100 in TBS) was applied instead. Sections were washed again in PBS for NeuN/DAPI- and Nestin/PCNA/DAPI-staining, and in TBS for NeuroD1/PCNA-staining before the primary antibody was applied overnight at 4°C.

Primary antibody, monoclonal mouse-anti-rat NeuN IgG (Merck Millipore, cat. no. MAB377, Darmstadt, Germany) diluted 1:200 in antibody diluent (Zymed Laboratories, San Francisco, CA, USA) or polyclonal goat anti-rat Nestin IgG (R&D, AF2736, Minneapolis, USA) diluted 1:20 in antibody diluent and monoclonal mouse anti-rat PCNA IgG (abcam, ab29, Cambridge, UK) diluted 1:1,000 in antibody diluent, or monoclonal mouse anti-rat NeuroD1 (abcam, ab60704) diluted 1:200 in antibody diluent and polyclonal rabbit anti-rat PCNA (abcam, ab152112) diluted 1:50 in antibody diluent was applied to each slide.

For NeuN/DAPI-staining sections were washed three times in PBS before the fluorescein-conjugated secondary antibody goat anti-mouse-IgG Alexa Fluor 488 (Thermo Fisher Scientific, Waltham, MA, USA, A11029, Rockford, IL, USA) diluted 1:200 in carrier solution was applied for 60 min in darkness at room temperature.

For Nestin/PCNA/DAPI- staining sections were washed three times in PBS before donkey anti-goat Alexa Fluor 488 (Thermo Fisher Scientific, Waltham, MA, USA, A11055) diluted 1:200 in antibody diluent was applicated for 4 h. Sections then were washed three times in PBS and goat anti-mouse Alexa Fluor 594 (Thermo Fisher Scientific, Waltham, MA, USA, A11032) diluted 1:200 in antibody diluent applicated for 60 min.

For NeuroD1/PCNA/DAPI- staining sections were washed three times in TBS before goat anti-mouse Alexa Fluor 488 (Thermo Fisher Scientific, Waltham, MA, USA, A11029) diluted 1:200 in antibody diluent was applied for 2 h. The sections were again washed three times in TBS and goat anti-rabbit Alexa Fluor 594 (Thermo Fisher Scientific, Waltham, MA, USA, A11037) diluted 1:200 in antibody diluent was applied for 60 min. Aqueous 4′,6-diamidino-2-phenylidole (DAPI; Sigma-Aldrich, #32670, Taufkirchen, Deutschland) 1:1,000 was applied for 10 min. After washing three times in PBS for NeuN/DAPI- and Nestin/PCNA/DAPI-staining, and three times in TBS for NeuroD1/PCNA/DAPI-staining, the sections were mounted and stored overnight at 4°C.

Images were acquired blinded on Keyence compact fluorescent microscope BZ 9000 with BZ-II Viewer software (Keyence, Osaka, Japan) using 10x objective lenses and individual files stitched automatically for each RGB color. Pictures were taken with the same exposure time and contrast/brightness parameters. Imaging files were analyzed and quantified in Adobe Photoshop software (Adobe Photoshop CS3 Extended). Double- (NeuN/DAPI) and triple-labeled (Nestin or NeuroD1/PCNA/DAPI) images in the DG were quantified by first outlining the hilus, GCL, and SGZ as a region of interest (ROI) at 10× magnification in Adobe Photoshop using DAPI to initially identify the cell-dense GCL. The area of the complete hilus, GCL, and SGZ of the DG, with an imaginary cut at the beginning of CA3, was counted manually for each staining up to four sections per animal separately. For multi-channel images, distinct channels were overlaid using the “Merge Channel”-function (overlaid images are indicated as “merge” in figures). To count the co-labeled positive cells, the different RGB channels were used overlapping. For each quantified marker, two different investigators for reproducibility repeated counts. The fluorescence signal for single reactivity and co-localization of immunoreactivity was counted individually using the markers function in the Adobe Photoshop software at 40× magnification. Mean values per sample were calculated by averaging the values of all sections of the same animal and were used to compare the cell counts of neuronal marker of GABA receptor antagonist treated animal vs. or control animals. For the representative imaging of immunohistological stainings, a background minimization with black balance identical for all images was performed.

### Statistical Analyses

Box and whisker plots represent the interquartile range (box) with the line representing the median, while whiskers show the data variability outside the upper and lower quartiles. Groups were compared using one-way analysis of variance (ANOVA), based on a partially non-Gaussian distribution with the Kruskal–Wallis test or based on the assumption that groups do not have equal variances with the Brown-Forsythe test. Depending on which ANOVA test was used, multiple comparisons of means were carried out using Bonferroni’s, Dunn’s, or Dunnett’s T3 *post hoc* test. A *p* value of <0.05 was considered significant. All graphics and statistical analyses were performed using the GraphPad Prism 8.0 software (GraphPad Software, La Jolla, CA, USA).

## Results

### GABA_B_ Receptor Antagonist Reduces Intermediate Progenitor Cells

To investigate the effect of GABAergic signaling on early progenitor cells at the beginning of neuronal cell lineage development, we stained for Nestin. This marker is expressed by NSCs (Nicola et al., 2015). The GABA_B_ receptor antagonist CGP administered in a dose of 2 mg/kg led to a decreased number of Nestin+ cells at the DG. The proliferating Nestin+/PCNA+ double-stained cells were significantly diminished in animals treated with the highest dose of GABA_B_ receptor antagonist CGP (10 mg/kg). The administration of the GABA_A_ receptor antagonist DMCM, in contrast, did not significantly alter the number of Nestin+ or Nestin+/PCNA+ double-stained cells (Figures 1A,B). The expression of the NSC marker *Pax6* was reduced in CGP-treated (10 mg/kg) animals. Multipotent NSCs, besides their neuronal qualities, also have astrocytic properties and thus express GFAP and Scl1a3 (DeCarolis et al., 2013; Berg et al., 2018). The expression of those astrocytic markers, *GFAP* and *Scl1a3*, was unchanged by either GABA receptor blockade (Figure 1C). By the expression of Hes5 and Sox2, NSCs inhibit neuronal differentiation and maintain their crucial multipotency and proliferation capacity (Pérez-Domínguez et al., 2018). The blockade of GABA receptors did not interfere with the expression of *Hes5* and *Sox2*.

### GABA_B_ Receptor Blockade Weakens Number and Proliferation of Intermediate Progenitor Cells

Ongoing neuronal differentiation of NSCs’ progeny leads to intermediate progenitor cells of type 2a. Those still dividing Nestin+ type-2a cells express Ascl1, a transcription factor sufficient to induce neuronal differentiation (Vasconcelos and Castro, 2014). We found decreased expression of *Ascl1* in CGP-treated animals. Ngn2 is another transcription factor up-regulated in type-2a cells (Pérez-Domínguez et al., 2018). *Ngn2* expression was not affected by the administration of GABA receptor antagonists (Figure 2C). The neuronal fate choice of intermediate progenitor cells leads to decreasing expression of Nestin while type-2b cells and neuroblast-like type-3 cells become NeuroD1 positive (Nicola et al., 2015). NeuroD1+ cells were reduced in all groups treated with CGP, indicating that intermediate progenitor cells at the differentiation stages of type-2b cells and neuroblast-like type-3 cells are especially affected by the GABA_B_ receptor blockade. The highest dose of CGP with 10 mg/kg also led to reduced proliferation of NeuroD1+/PCNA+ double-stained cells (Figures 2A,B). In line with these results, qPCR showed a significant reduction of *NeuroD1* at RNA gene expression level in CGP-treated animals. The expression of *Tbr2*, a broader marker for all type-2 cells, was not altered by CGP. DMCM-treatment on the contrary did not affect the number of NeuroD1+ and NeuroD1+/PCNA+ cells and did not change *Ascl1* or *NeuroD1* expression, but led to an increased *Tbr2* expression.

### Postmitotic Neurons Are Less Affected by GABA Receptor Blockade

NeuN staining (Vieira et al., 2018) identified postmitotic neurons. The number of postmitotic neurons in the DG was not significantly affected by the administration of GABA receptor antagonists (Figures 3A,B). The expression of *Tbr1*, however, another marker for postmitotic neurons, was reduced in CCP-treated animals and increased in DMCM-treated ones. *NeuroD2*, a third marker for postmitotic neurons, showed decreased expression after CGP-treatment and was not affected by DMCM. *Prox1*, involved in granule cell maturation (Lavado et al., 2010), was not altered by the administration of GABA receptor antagonists (Figure 3C).

### GABA_B_ Receptor Antagonists Affected Hippocampal Proliferation Capacity

The highly regulated ability of precursor cells to proliferate and self-renew is pivotal for the functioning of hippocampal neurogenesis up to adult age (Berg et al., 2018). In order to investigate the dependency of hippocampal proliferative capacity on GABA receptor blockade, slices were stained for the endogenous proliferation marker PCNA. The number of proliferating and therefore PCNA+ cells increased after treatment with 2 mg/kg CGP, while it was not altered in DMCM-treated groups (Figures 3A,B). It should be noted that despite a significant difference in the CGP-treated animals compared to the control, the variance is very high. Additionally, we observed an increased expression of *CycD2* in CGP-treated animals.

### GABA_B_ Receptor Blockade Reduces Expression of Neurotrophins

Neurotrophins play important roles in defining the hippocampal neurogenic niche (Faigle and Song, 2013). Brain-derived neurotrophic factor (BDNF) affects structural plasticity, dendritic spine growth, and has long–term effects on LTP and learning (Leal et al., 2015; Gonçalves et al., 2016). Nerve growth factor NGF as well as neurotrophin 3 NT-3 are involved in the regulation of synaptic plasticity (Leal et al., 2015). All tested neurotrophic factors, *BDNF*, *NGF*, and *NT-3*, were downregulated in rats treated with CGP while DMCM did not alter the expression of any of these neurotrophic factors (Figure 4).

Briefly, we observed a reduction of NPC, especially of type-2b and type-3, caused by GABA_B_ receptor blockade. In addition, the proliferation of progenitors at these stages in hippocampal neurogenesis was diminished. Gene expression analysis revealed a reduced expression of multiple transcription factors involved in neuronal fate choice (see schematic diagram in Figure 5). After GABA_A_ receptor blockade, in contrast, there was no significant impairment of neurogenesis observed on a cellular level and changes in RNA-expression affected only *Tbr 1* and *Tbr 2*, leading to an overexpression of these factors.

## Discussion

In the present study, we investigated the effects of a GABA_A_ and GABA_B_ receptor antagonist on postnatal hippocampal neurogenesis in newborn rats, during the developmental depolarizing-to-hyperpolarizing switch in response to the neurotransmitter GABA (Ben-Ari, 2018). Our data suggest that antagonizing GABA_B_ receptor activity impaired the progression of neural progenitors to mitotic immature neurons, reduced the expression of neurotrophins, and increased the proliferation capacity. In contrast, antagonizing GABA_A_ receptors did not affect postnatal hippocampal neurogenesis at a cellular level.

GABA is one of the most abundant neurotransmitters in the central nervous system. GABAergic neurons are the main source of GABA; additionally, synthesis occurs in glial cells (Angulo et al., 2008; Héja et al., 2012). The subtypes of GABA_B_ receptors, GABA_B1a/b_ and GABA_B2,_ were shown to be located both pre- and postsynaptically, indicating a more modulatory function in the developing brain (López-Bendito et al., 2004; Evenseth et al., 2020). Various experimental models suggest that GABA_B_ receptor antagonists modulate hippocampal-linked learning, memory, and behavior (Cryan and Kaupmann, 2005; Heaney and Kinney, 2016).

During neuronal lineage development, intermediate progenitor cells have been described to successively express a cascade of transcription factors starting with Pax6 expression in type-1 and type-2a cells, and further involving Ngn2, Tbr2, NeuroD1, Tbr1, and Prox1, which is expressed from the stage of type-2b cells onwards (see schematic overview in Figure 5; Gonçalves et al., 2016; Pérez-Domínguez et al., 2018; Hevner, 2019). First, GABA_B_ receptor blockade reduced the total Nestin+ and proliferating Nestin+ cells in the DG, depending on concentration. Nestin is widely used as a marker for proliferating neuroepithelial and progenitor cells as well as some astrocytes in the hippocampus (Von Bohlen und Halbach, 2011; Wilhelmsson et al., 2019). Further, Nestin is essential for the survival and self-renewal of NSC (Park et al., 2010). Quiescent type-1 NSC and active NSC differ in frequency of proliferation, while both cell types share the characteristic expression of the glial cell marker GFAP and neural progenitor marker Nestin (Lugert et al., 2010). Neither *GFAP* nor the astrocytic markers *SCL1a3/GLAST* were impaired in gene expression by the antagonization of the GABA_B_ receptor. Sox2-expressing cells in the SGZ generate differentiated and identical cells, indicating their multipotent properties and importance for self-renewal (Suh et al., 2007). Similar to Sox2, Hes5-positive cells specifically inhabit the SGZ, with the property of NPC in the neurogenic niche (Lugert et al., 2010). Both transcription factors of type-1 cells, *Sox2* and *Hes5*, were not affected by the blockade of GABA. This is in line with the thesis that type-1 NSCs are not affected as a whole, while the proliferating subpopulation of type-1 NSC depends on GABAergic signaling.

It is interesting that *Pax6*, classifying newly born cells from the differentiation state in SGZ and regulating self-renewal of NSC (Maekawa et al., 2005), was significantly reduced by the performed GABA_B_ receptor blockade. The impact on Nestin+ NSC and *Pax6* expression suggests that GABA_B_ receptor-mediated GABA signals may be central in determining the neuronal fate and proliferation of NSCs. NSCs self-renew to generate asymmetrically a new daughter NSC and a daughter NSC with properties of intermediate NPC (type-2 cells; Gonçalves et al., 2016). The maintenance of pluripotency in NPC by Sox2 is well-defined (Favaro et al., 2009). Sox2 binds to the regulatory regions of a number of genes involved in neuronal differentiation (Lodato et al., 2013), including proneural genes such as Ascl1 (Vasconcelos and Castro, 2014) and NeuroD1 (Kuwabara et al., 2009). The substitution of Sox2 by neurogenic signals at the regulatory promotor regions is required for activation of proneural genes and therefore downregulation of Sox2 a precondition for further neuronal differentiation (Kuwabara et al., 2009). GABA antagonists did not interfere with the level of Sox2 expression in the postnatal DG.

One step further in neuronal differentiation, changes caused by GABA receptor blockade were detectable. Mediators of intermediate progenitor cells, *Ascl1*, *NeuroD1* as well as proliferating NeuroD1+ cells were significantly decreased under GABA_B_ receptor blockade. Ascl1 is a proneural transcription factor and is expressed in mitotic NPC (Uda et al., 2007). The transcription factor NeuroD1, which may act as a neuronal determination gene, is expressed in the middle and late stages of NPC (Von Bohlen und Halbach, 2011). Interestingly, Ascl1 is able to play two opposing roles. On the one hand, it promotes proliferation and on the other supports cell-cycle exit and differentiation (Andersen et al., 2014). Ascl1 enables the transcription of multiple proneuronal target genes (Vasconcelos and Castro, 2014). A decreased expression of Ascl1 after GABA_B_ receptor blockade might explain the observed delay in NPC differentiation. This does not necessarily lead to decreasing proliferation *per se*. Although the proportion of mitotic maturing neurons (NeuroD1+/PCNA+ cells) was reduced, the overall proliferation capacity (PCNA+ cells, *CycD2*) was induced. This could be due to Ascl1’s interaction with Notch target genes in neural precursor cells *via* downstream mediators (Andersen et al., 2014), similar to Hes5 (Lugert et al., 2010). Hes proteins activated *via* the Notch signaling pathway act as repressors of proneural transcription factors. Due to their self-regulation and short half-lives, the cellular expression of Hes proteins is not constant and thus leads to fluctuating expression of downstream target genes, such as Ngn2 and Ascl1 (Lugert et al., 2010; Vasconcelos and Castro, 2014). Expressions that can be changed in this way could promote proliferation, while steady conditions lead to the progression of differentiation.

Current measurement technology at a certain point is not sufficiently capable of detecting oscillating expressions of these mediators. However, our findings support the hypothesis that NeuroD1, which is directly regulated by Pax6 (Thakurela et al., 2016), and Ascl1 are significantly reduced in expression, while proliferation is increased. Typically, NeuroD1 expression peaks in early neuroblasts/type-2b and type-3 cells. Diminished NeuroD1 expression seems to be partly responsible for the stagnation of differentiation and progression of neurogenesis (Gao et al., 2009).

A possible explanation for the observed general boost in proliferation capacity beyond the transcriptional regulation of hippocampal neurogenesis might be GABAergic signaling itself. GABAergic signals through GABA receptors as well as the loss of GABA_A_ and GABA_B_ receptor subtypes were described to increase proliferation of NSC in the adult DG (Song et al., 2012; Giachino et al., 2014). GABA_B_ receptor function is associated with suppressed proliferation of adult hippocampal neurogenesis (Felice et al., 2012). The impact of GABA_B_ receptor blockade already in postnatal development with excitatory GABA on NPC associate transcription factors suggests that GABA_B_ receptor-mediated signals may be important for initiating transcription of proneural mediators and generate mitotic neuroblasts.

Nevertheless, mitotic immature neuroblasts/type-2b cells express Tbr2 (Hodge et al., 2012). Before the neuroblasts exit the cell cycle to become mature granular neurons, they start expressing Tbr1, Prox1, and NeuroD2 (Gonçalves et al., 2016). Downregulation of Tbr2 is the precondition for NPC to differentiate into postmitotic immature neurons and this coincides with upregulation of Tbr1, which is strongly associated with new mature neurons (Hodge et al., 2012). Late neuroblasts/type-3 cells and postmitotic granular neurons express the transcription factor Prox1 (Li et al., 2009). NeuroD2 induces a neural phenotype, while its possible involvement in terminal differentiation is still being investigated (Ravanpay et al., 2010). Considering the crucial tasks of these transcription factors for final neurogenic termination, a significant downregulation of gene expression would suggest a reduction in the number of mature neurons. Controversially, GABA_B_ receptor antagonization sustainably reduced the expression of relevant transcription factors (*Prox1, Tbr1, NeuroD2*), but did not affect the NeuN+ neurons in DG.

Prox1 was applied as a dentate granule neuron lineage marker (Rubin and Kessaris, 2013). Several studies (Lavado et al., 2010; Iwano et al., 2012) suggested Prox1 to act pleiotropically and modulate multiple targets as well as signaling pathways possibly through dysregulation of Notch signaling. Postnatal deletion as well as a conditional knockout of Prox1 in mice results in decreased counts of NPC subtypes (type-2a/b cells) and NSC/ type-1 cells (Lavado et al., 2010). Further, overexpression of NeuroD1, but not of either Ngn2 or NeuroD2, was able to drive the exclusive production of new neurons in the adult hippocampus (Richetin et al., 2015). A milieu dominated by the differentiation-driving effects of NeuroD1 might overlay the stage-specific action of NeuroD2. Particularly since NeuroD2 is required for the survival of hippocampal neurons (Olson et al., 2001), one might expect apoptosis-associated mediators to increase; however, in our study neither the expression profiles of apoptosis-associated mediators, effector caspase 3 (*Casp3*), and apoptosis-inducing factor (*AIF*), nor the total DAPI+ cell counts showed changes after switching off GABA receptor signaling compared to the control animals (see Supplementary Figures 1A,B).

The temporal delay in the differentiation of the NPC in our model of GABA_B_ receptor blockade is in line with a hypothesis formulated by Lugert et al. (2012). Instead of the amplification of type-2a intermediate progenitors as seen previously, though, they proposed that divisions of neural stem cells and early neuroblasts drive DG neurogenesis (Lugert et al., 2012). Mitotic NSCs in the DG provide neuronal progenitor expansion and initiate neurogenic differentiation, a process requiring more than 3 weeks under homeostatic conditions. After the initiation of neurogenesis, cells remain at the immature NPC developing stage for some weeks. In this phase, neuroblasts adapt to physiological or pathophysiological stimuli that may affect their maturation and differentiation. The NPC population reacts to specific noxae and, if required, rapidly initiates the production of many neurons (Dranovsky et al., 2011; Lugert et al., 2012). The hypothesis that NPCs remain in an immature state for a long period and retain their ability to expand neuronal cells and continue neurogenesis in order to quickly provide missing neurons when needed could support our observation of an unchanged population of mature neurons after GABA receptor blockade despite massive impairment of intermediate neurons. A hypothetical subsequent effect on the proportion of mature neurons resulting from a reduced differentiation capacity of maturing intermediary neurons did not occur at time P15 (see Supplementary Figure 2).

The described impact of transcription factors associated with mature granular neurons suggests that GABA_B_ receptor-mediated GABA signals might be important for the maintenance of the NPC cell pool by the initiation of pleiotropic interactions. Since glial markers were not influenced by GABA_A_ or GABA_B_ receptor antagonization, only the neural line seems to be affected. Interestingly GABA_A_ receptor blockade did not lead to any changes similar to those caused by GABA_B_ receptor blockade. Instead, it resulted in an overexpression of *Tbr2* and *Tbr1*. There seem to be essential differences regarding the influence of GABA and its mediation *via* the respective receptors. Considering the GABA receptor density, a significant increase of GABA_A_ receptor occurs between P10 and P20, while the GABA_B_ receptor density peaks between P0 and P10 at P5 (Behuet et al., 2019). Due to the animal model we used, an effect of GABA blockade *via* the GABA_B_ receptor seems more probable. GABA-mediated effects *via* the GABA_A_ receptor require further investigation.

Intrinsic factors including neurotransmitters (Catavero et al., 2018), hormones (Triviño-Paredes et al., 2016), and neurotrophic factors (Lee et al., 2002) influence hippocampal neurogenesis. Neurotrophic factors, such as NT-3, NGF, and BDNF, are key players in the stimulation of NSC proliferation and differentiation (Ding et al., 2013). Interestingly, GABA_B_ receptor peak coincides with the time course of synaptogenesis, which is regulated by neurotrophins such as BDNF (Gaiarsa and Porcher, 2013). Coordinated effects of GABA and neurotrophins are important for the long–term survival of newborn neurons (Vilar and Mira, 2016). Waterhouse et al. (2012) suggested a reciprocal interaction between neurotransmitters and neurotrophic factors and showed that BDNF promotes progenitor cell differentiation and maturation by enhancing the release of GABA in the SGZ, resulting in increased differentiation and maturation of progenitor cells. Glial cells and GABAergic neurons are not sources for the secretion of neurotrophins themselves. BDNF is provided by pyramidal neurons or mossy fibers of hippocampal granular cells (Danzer and Mcnamara, 2004). BDNF is a crucial regulator of synapse development (Cohen-Cory et al., 2010) and shapes the development of neuronal circuits, as well as the construction of inhibitory connections throughout life (Gottmann et al., 2009). Similar findings were reported for NGF and NT-3, both involved in several critical processes in the developing brain (Shimazu et al., 2006; Frielingsdorf et al., 2007). We found that the inactivation of metabotropic GABA_B_ receptors downregulates the transcription of *BDNF*, *NGF*, and *NT-3*. Currently, studies on the effects of GABA_B_ receptor blockade on the neurotrophic response are limited. Overexpression of BDNF in hippocampal cultures results in an earlier maturation of inhibitory synapses by inducing both presynaptic and postsynaptic structural and functional modifications to enhance GABAergic transmission (Vicario-Abejón et al., 1998). Badurek et al. (2020) show that early disruption of Trkb signaling, the receptor for BDNF, from immature mouse hippocampal dentate granule cells affects the integration and maturation of newly formed DGCs in the hippocampal circuitry, and drives a premature shift from depolarizing to hyperpolarizing GABAergic actions. Conditional NT-3 knockout in mice goes along with an impairment of neuronal differentiation (Shimazu et al., 2006). Intracerebroventricular treatment with NGF increases hippocampal neurogenesis and enhances the survival of new neurons in young and aged rats (Frielingsdorf et al., 2007). Likewise, following GABA_B_ receptor activation and regulated secretion of BDNF increases the number of postsynaptic GABA_A_ receptor subunits (Kuczewski et al., 2011). This implies complex interactions between GABA_B_ receptor activation and neurotrophins to contribute to the functional and structural maturation of the developing hippocampus. A decreased neurotrophic response following GABA_B_ receptor antagonization appears to be consistent with the stagnant maturation of NPC. Neurotrophins may have important roles in hippocampal neurogenesis and developmental plasticity (Porcher et al., 2018). Imbalance in neurotransmission seems to lead to the reduction of neurotrophin transcription; therefore, further investigations on the precise mechanisms of the relationship between neurotrophic growth factors and GABAergic transmission are required.

Taken together, GABA influences the development and functioning of the GABAergic network mainly *via* GABA_B_ receptor during the first 10 postnatal days. As a result of the limited signal transduction of GABA *via* the GABA_B_ receptor, impairment of neurogenesis advanced, which did not affect the final termination to mature neurons, and diminished neurotrophin signals. This could imply that although antagonization of GABA_B_ receptors recruited quiescent cells to the active proliferative stem cell pool, progression towards terminal differentiation was not induced. This is in line with Giachino et al.’s (2014) proposal that GABA_B_ receptor activity controls the number of proliferating NPC in the adult hippocampus. In sum, GABA and the GABA_B_ receptors’ transmission affect neural stem cell and progenitor cell proliferation in the developing hippocampus.

The involvement of GABA receptors in neurogenesis was linked to the modulation of cognitive processes, like memory formation, executive function, learning, and intelligence. Imbalances during GABAergic transmission in neuronal circuits, such as drugs or oxygen, can affect these vulnerable phases of brain development and disrupt homeostatic control (Vertkin et al., 2015; Friedman and Kahen, 2019). Failure in neuronal homeostasis has been linked to pathophysiological mechanisms of various brain disorders (Marín, 2012; Kim and Yoon, 2017), like autism, hyperactivity, inattention, social and emotional incompetence, which are also associated with preterm birth (Fatemi et al., 2009; Arpino et al., 2010; Hashemi et al., 2017). For a deeper understanding of postnatal brain development and improving the understanding of GABA signaling in correspondence to preterm birth, further analysis will be required to establish which cues regulate the various stages of neurogenesis beyond NSC expansion, NPC proliferation, and maturation as well as the survival of newly formed neurons. These may open new therapeutic strategies to alleviate behavioral impairments and neurological disorders, perhaps in part as sequela of early derangement in GABAergic systems.

### Limitations of the Study

Altogether, these observations point to GABA as one of the major players in the early formation of neuronal circuits in the developing brain. Since GABA_B_ receptors are expressed in hippocampal progenitor cells as well as throughout the adult neurogenic lineage, it is possible that antagonization of GABA receptor signaling affects non-hippocampal neuronal cells. For gene expression analysis, we used the complete hemisphere and removed the olfactory bulb and cerebellum. The data collected cannot be adequately and exclusively associated with the hippocampus and we are aware that the transcriptional markers are also expressed in other brain regions of the developing brain (Rodier, 1980; Rice and Barone, 2000; Lavado and Oliver, 2007; Bedogni et al., 2010; Hsieh, 2012; Miyoshi et al., 2015). The main proliferating niche during the phase of rapid brain growth is the DG (Stefovska et al., 2008). A study by Stefovska et al. (2008) showed in the developing brain (P0 to P15), that the *in vivo* modulation of GABA receptors changes the proliferation capacity in different brain regions, such as cortical sections, thalamus, and in the DG (SGZ and granular cell layer) to the same extent. Not only newly generated neurons in the proliferative neurogenic niches of the immature brain express GABA receptors, so that an impairment after systemic administration of GABA antagonists or agonists can act on different reaction pathways, on different cell types and can affect cells practically in all parts of postnatal brain. Nevertheless, postnatal cell proliferation is age-dependent and most pronounced in the cerebellum and the SGZ of the DG. Outside the cerebellum and DG, proliferating new neurons become non-neuronal cells, like glia cells. The neurotransmitter GABA constitutes a developmental signal during stages of embryonic neurogenesis, progenitor proliferation, neuronal migration, and neurite outgrowth (Wang and Kriegstein, 2009; Xing and Huttner, 2020). Differences in the transcription of neurogenesis-associated genes and specifically neuronal lineage associated cells are then less expected in the whole brain RNA or protein extract (if removal of olfactory bulb and cerebellum) as all are affected to the same extent. Significant change of transcript levels may prove the influence of GABAergic interruptions in the neonatal brain, including possible already migrated cells, in whole hemisphere homogenate. Further studies could advance our observed data with region-specific transcription analyses in the developing brain.

## Data Availability Statement

The original contributions presented in the study are included in the article/Supplementary Material, further inquiries can be directed to the corresponding author.

## Ethics Statement

The animal study was reviewed and approved by LAGeSo, Berlin, Germany, approval number G-0075/18.

## Author Contributions

TScheuer and SE conceived the ideas. TScheuer designed the experiments. SE, CG, and TScheuer executed the experiments. CG and SE wrote the first draft of the manuscript and approved the final draft. TScheuer and SE performed the animal studies. CG performed and analyzed immunohistological staining and qPCR. TSchmitz and CB revised the manuscript and contributed to the critical discussion. All authors have contributed to the article and approved the submitted version.

## Conflict of Interest

The authors declare that the research was conducted in the absence of any commercial or financial relationships that could be construed as a potential conflict of interest.

## Publisher’s Note

All claims expressed in this article are solely those of the authors and do not necessarily represent those of their affiliated organizations, or those of the publisher, the editors and the reviewers. Any product that may be evaluated in this article, or claim that may be made by its manufacturer, is not guaranteed or endorsed by the publisher.

## Supplementary Material

SUPPLEMENTARY FIGURE 1 Quantification of **(A)** DAPI positive cells in sum of the DG of control animals (100% white bars), DMCM hydrochloride (DMCM) in doses of either 2 μg/kg, 10 μg/kg or 50 μg/kg, and CGP 35348 in doses of either 0.4 mg/kg, 2 mg/kg or 10 mg/kg treated rat pups at P11. Data are expressed relative to the control group as mean ± SEM of *n* = 10 each group. The 100% values are 594.1 cell counts (Brown-Forsythe test). Expressions of **(B)** *AIF* and *Casp3* are not affected by the application of DMCM or CGP. The relative mRNA expressions of markers were measured by quantitative real-time PCR in rat brain homogenates with DMCM 50 μg/kg (gray bars) or CGP 10 mg/kg (black bars) application relative to control (white bars). Bars represent the relative mRNA quantification based on internal standard *HPRT*. Data shown as mean ± SEM, *n* = 9–10 (one-way ANOVA).

SUPPLEMENTARY FIGURE 2 Representative hippocampal paraffin sections **(A)** of control animals, DMCM hydrochloride (DMCM) in dose of 50 μg/kg and CGP 35348 in dose of 10 mg/kg treated rat pups at P15 co-labeled with DAPI and NeuN. Application of GABA receptor antagonists did not affect cell counts for postmitotic NeuN+ neurons at the DG. Quantification of **(B)** NeuN positive cells in sum of the DG in comparison to control group (100% white bars). Data are expressed relative to the control group as mean ± SEM of *n* = 10 each group. The 100% values are for NeuN+ 187.3 cell counts (one-way ANOVA).
