# Supplementary figures and images for: GABAB Receptor-Mediated Impairment of Intermediate Progenitor Maturation During Postnatal Hippocampal Neurogenesis of Newborn Rats
Source: Front Cell Neurosci. 2021 Aug 6;15:651072. doi: 10.3389/fncel.2021.651072 (PMC8377254; doi:10.3389/fncel.2021.651072)

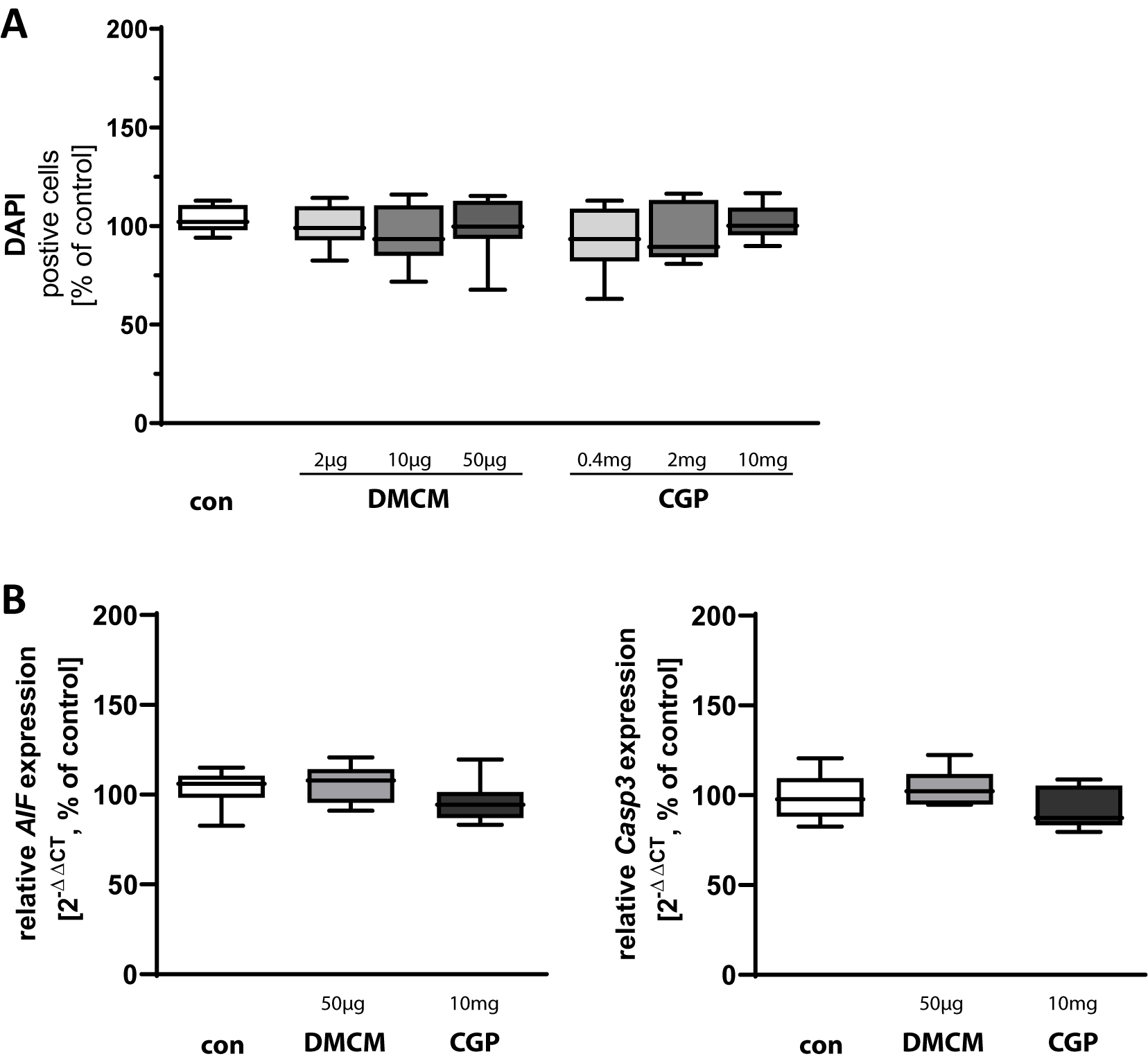

Supplement: SUPPLEMENTARY FIGURE 1 — Quantification of (A) DAPI positive cells in sum of the DG of control animals (100% white bars), DMCM hydrochloride (DMCM) in doses of either 2 μg/kg, 10 μg/kg or 50 μg/kg, and CGP 35348 in doses of either 0.4 mg/kg, 2 mg/kg or 10 mg/kg treated rat pups at P11. Data are expressed relative to the control group as mean ± SEM of n = 10 each group. The 100% values are 594.1 cell counts (Brown-Forsythe test). Expressions of (B) AIF and Casp3 are not affected by the application of DMCM or CGP. The relative mRNA expressions of markers were measured by quantitative real-time PCR in rat brain homogenates with DMCM 50 μg/kg (gray bars) or CGP 10 mg/kg (black bars) application relative to control (white bars). Bars represent the relative mRNA quantification based on internal standard HPRT. Data shown as mean ± SEM, n = 9–10 (one-way ANOVA). [file Image_1.TIF]

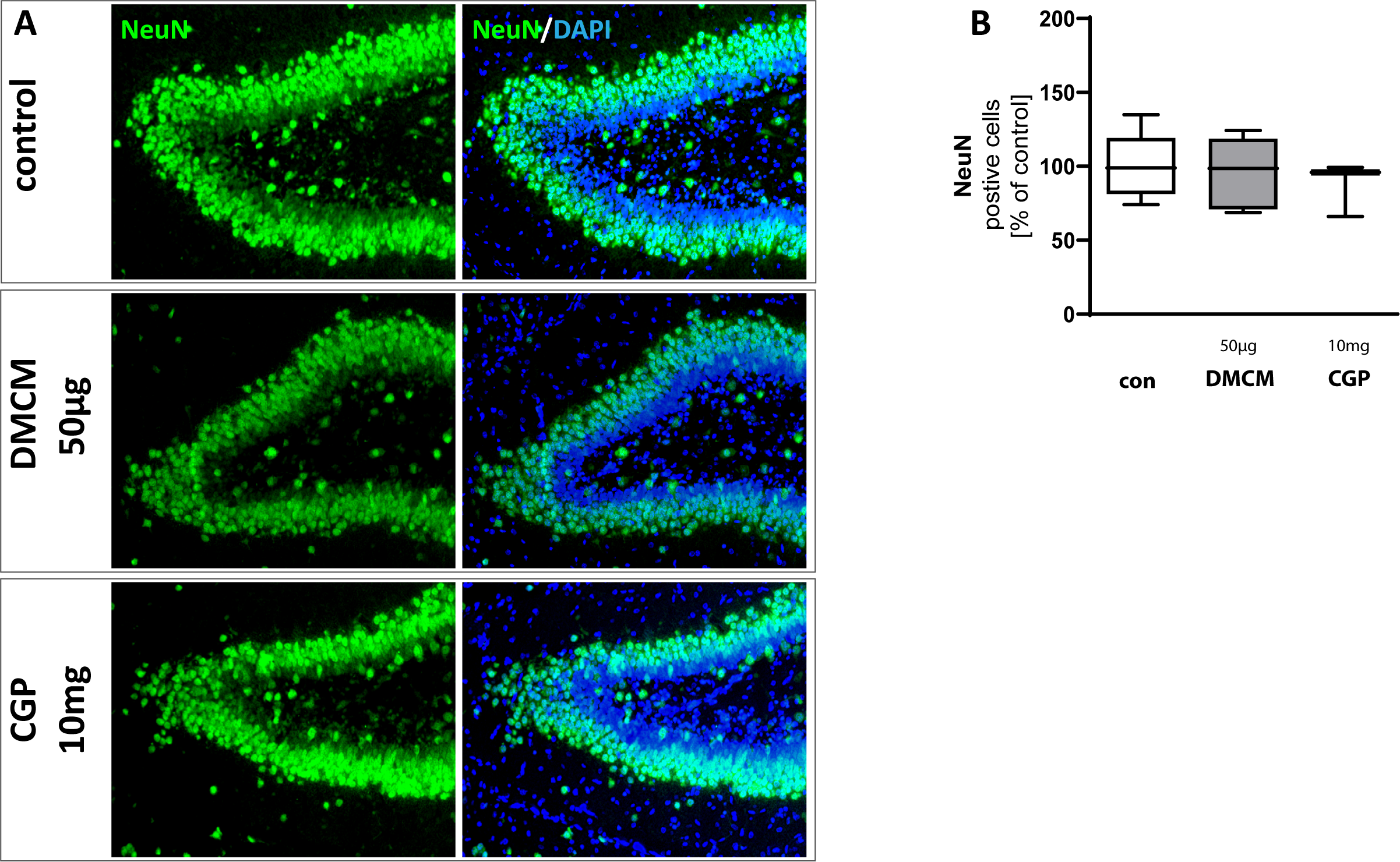

Supplement: SUPPLEMENTARY FIGURE 2 — Representative hippocampal paraffin sections (A) of control animals, DMCM hydrochloride (DMCM) in dose of 50 μg/kg and CGP 35348 in dose of 10 mg/kg treated rat pups at P15 co-labeled with DAPI and NeuN. Application of GABA receptor antagonists did not affect cell counts for postmitotic NeuN+ neurons at the DG. Quantification of (B) NeuN positive cells in sum of the DG in comparison to control group (100% white bars). Data are expressed relative to the control group as mean ± SEM of n = 10 each group. The 100% values are for NeuN+ 187.3 cell counts (one-way ANOVA). [file Image_2.TIF]
